# Navigating ACL Injuries Amid the COVID-19 Pandemic: A Retrospective Analysis

**DOI:** 10.7759/cureus.47857

**Published:** 2023-10-28

**Authors:** Saphalya Pattnaik, Majd Algharibeh, Ahmadreza Zarifian, Gur Aziz Singh Sidhu, Jacinder Chahal, Shahid Punwar

**Affiliations:** 1 Trauma and Orthopaedics, University Hospital Lewisham, London, GBR; 2 Trauma and Orthopaedics, University Hospitals of Derby and Burton NHS Foundation Trust, Burton-on-Trent, GBR; 3 Trauma and Orthopaedics, Queen Elizabeth Hospital, London, GBR

**Keywords:** covid-19, orthopedic intervention, knee ligament injuries, anterior cruciate ligament (acl), mri imaging

## Abstract

Introduction

In the United Kingdom, musculoskeletal injuries represent a substantial proportion of primary care appointments, and within this category, acute knee injuries are prominent in accident and emergency department cases. Notably, diagnosing anterior cruciate ligament (ACL) injuries is a recognized challenge, often leading to extended diagnostic delays. The COVID-19 pandemic has significantly affected the management of musculoskeletal disorders, driven by reduced surgical capacities, creating a backlog of patients awaiting necessary surgical interventions. Delayed ACL reconstruction poses risks of prolonged knee instability and secondary injuries. To address these concerns, this study evaluates the impact of COVID-19 on the timeline from ACL injury diagnosis to MRI and surgical intervention, with a specific focus on an internal acute knee clinic pathway designed to expedite the evaluation, diagnosis, and management of soft tissue knee injuries.

Methods

In this cross-sectional study, we retrospectively reviewed all the patients who underwent primary ACL reconstruction from January 2019 to November 2022 in a district general teaching hospital (DGH). Besides demographic data of the patients, we recorded the dates of injury, primary presentation, first knee specialist review, knee MRI, and ACL reconstruction surgery, as well as the injury-to-surgery, injury-to-MRI, and MRI-to-surgery periods. Patients were divided based on the date of operation to pre- and post-COVID, and outcomes were compared to see the possible effects of COVID-19. Data were analyzed using a quantitative and qualitative test with a P < 0.05 significance level.

Results

Our cohort included 97 patients, and the mean age of patients was 30.6 years (17-53 years). The median time of injury-to-MRI was 46.5 days during the pre-COVID period, which decreased to 44 days in the post-COVID period (P = 0.596). The median time of injury-to-surgery was 287.5 days during the pre-COVID period, which increased to 289 days in the post-COVID period (P = 0.019). The median MRI-to-surgery duration was 200 days during the pre-COVID period, which increased to 225 days in the post-COVID period (P = 0.006). Around 35% of patients had an MRI prior to getting evaluated by a specialist.

Conclusion

The COVID-19 pandemic had a significant impact on the management of ACL injuries, with delays in elective knee clinics and surgery potentially leading to delays in the diagnosis and management of such injuries. However, our study showed that the delay from ACL injury to subsequent surgery actually reduced post-pandemic due to hospital-based acute knee pathway, which is particularly important in the context of associated meniscal injury that can worsen while patients wait for surgery.

## Introduction

In the United Kingdom, musculoskeletal injuries account for 30% of primary care appointments [1], with acute knee injuries accounting for approximately 5-8% of all acute injuries seen in the accident and emergency department [2,3]. Anterior cruciate ligament (ACL) injuries are widely recognized for being difficult to assess, and doctors frequently miss them during the initial assessment [4,5]. A study conducted by Bollen and Scott suggests that, on average, it took 21 months (about two years) to diagnose ACL injuries due to not being assessed by a knee ligament specialist [6].

Although orthopedic surgeons might not usually be considered front-line when treating patients during COVID-19, the burden of musculoskeletal disorders and injuries has been substantially affected by the pandemic, especially due to reduced surgical theater capacities [7]. Several studies indicate a 74% decline in arthroplasty and an 84% decrease in sports medicine cases in the United States, with a comparable decrease in arthroplasty and arthroscopy operations in the United Kingdom, producing a backlog of patients anticipating surgery [8,9].

A delay in ACL reconstruction may lead to a prolonged period of instability in the knee, potentially leading to meniscal injuries or further articular cartilage damage [10].

In line with the British Orthopedic Association Standard for Trauma (BOAST): Best Practice for the Management of ACL Injuries, urgent magnetic resonance imaging (MRI) should be facilitated and documented by a musculoskeletal physician [11]. Although there weren’t any set local guidelines in our hospital defining urgent MRI scans, the technicians usually try to get it done as soon as possible, depending on the urgency of the clinician’s request. Moreover, there is an absence of literature specifically addressing soft tissue injuries in the ambulatory setting.

It is critical to understand current evaluation methods and diagnostic pathways for patients with acute ACL injuries in order to create more effective diagnosis suggestions and assessment measures. We have a designated acute knee clinic pathway focused on enhancing the flow of soft tissue knee injuries for early evaluation, diagnosis, and management of such injuries. The purpose of this study was to assess the impact of COVID-19 on the time it takes from diagnosis of ACL injuries to MRI and surgical intervention with the provision of an internal acute knee clinic pathway.

## Materials and methods

Settings

In this cross-sectional study, we retrospectively reviewed patient records in a London teaching hospital during January-April 2023. The study was approved by the hospital’s clinical governance department and deemed exempt from ethical review as it was part of the departmental quality improvement.

Inclusion and exclusion criteria

The inclusion criteria included all patients who underwent primary ACL reconstruction surgery from January 2019 to November 2022 in our hospital.

The exclusion criteria included patients who chose to delay their management process or use private services, patients whose records were incomplete or missing, and those who required revision procedures.

Data gathering

**Figure 1:**
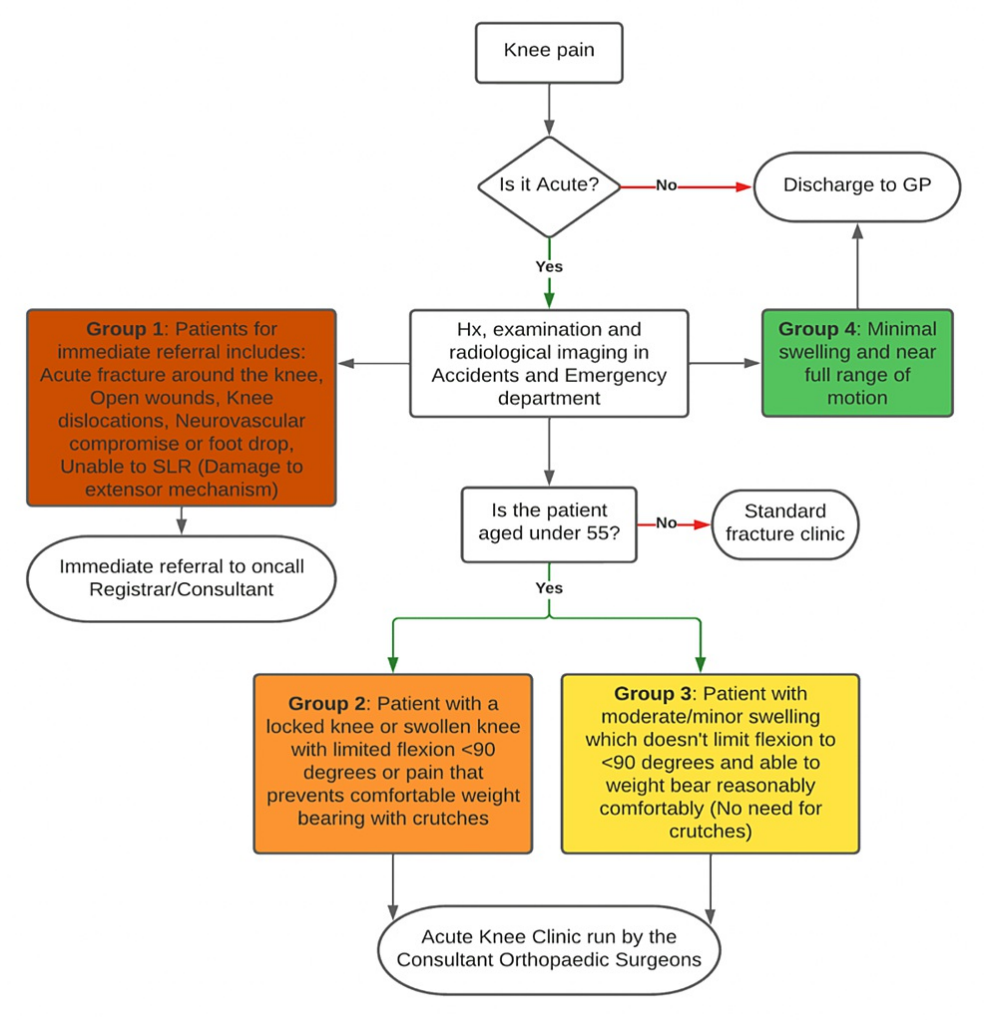
Local acute knee clinic pathway GP, general practitioner; SLR, straight leg raise test; Hx, history

The patients with new knee injuries were seen in an acute knee clinic (designed just prior to the pandemic) and streamlined for the operation via a process, as depicted in Figure 1. The provision of this knee clinic was brought into practice due to the ever-increasing demand related to trauma post-pandemic. Due to the emergence of COVID-19, all elective operations were canceled in our hospital for a period of six months in 2020. Based on the time of operations, we divided patients into “pre-COVID” and “post-COVID.” All operations done before March 13, 2020, were considered pre-COVID, and those performed on or after September 1, 2020, were considered post-COVID. The period between these dates had sporadic or completely canceled lists.

According to patient clinical notes, we recorded the dates of injury till surgery, primary presentation, first consultation with a knee specialist, MRI dates, and findings in a Microsoft Excel spreadsheet. We calculated the number of days, average, and median for injury-to-surgery, injury-to-MRI, and MRI-to-surgery periods.

Statistical analysis

Statistical analysis was done using the IBM SPSS Statistics, version 24.0 (IBM Corp., Armonk, NY). We used the one-sample Kolmogorov-Smirnov test to check data distribution and the Mann-Whitney U test to compare parameters between the two groups. All tests were run using P < 0.05 as statistically significant.

## Results

**Figure 2:**
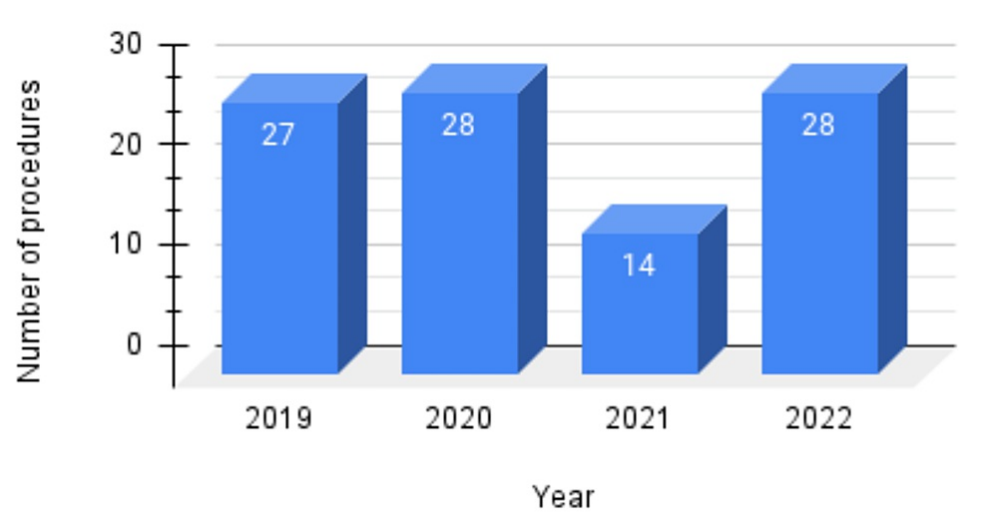
ACL reconstructions performed during 2019-2022 (N = 97) ACL, anterior cruciate ligament

Overall, 130 patients were studied, and outliers were excluded. The final study size was 97, and the mean age of the patients was 30.6 years. Figure 1 shows the distribution of ACL reconstructions during 2019-2022. The main outcomes, namely the injury-to-MRI, injury-to-surgery, and MRI-to-surgery duration, are compared between the two groups separately, as shown in Figure 2.

Injury-to-MRI

**Figure 3:**
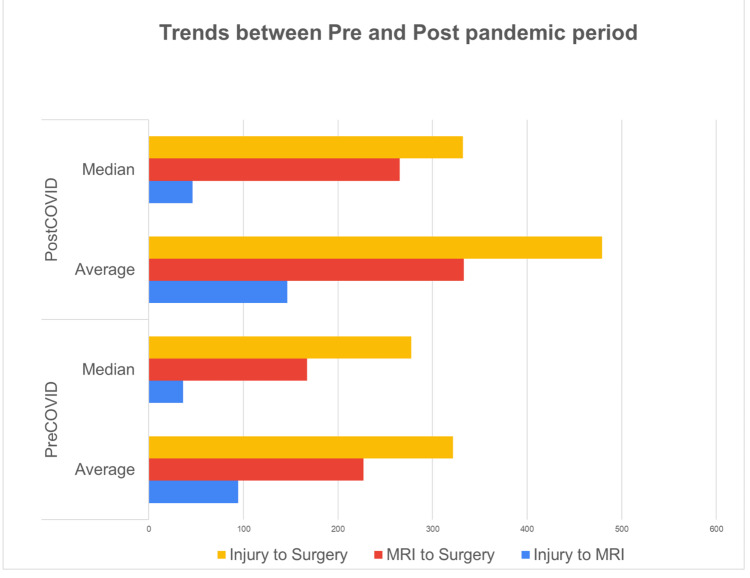
Trends in injury-to-MRI, MRI-to-surgery and injury-to-surgery compared between pre- and post-pandemic periods

The median time of injury-to-MRI was 46.5 days during the pre-COVID period, which decreased to 44 days in the post-COVID period. The analyses showed no significant difference between the two periods in this regard (P = 0.596) (Figure 3).

Injury-to-surgery

The median time of injury-to-surgery was 287.5 days during the pre-COVID period, which increased to 289 days in the post-COVID period. The difference between the two periods in this regard failed to make any statistical significance (P = 0.019) (Figure 3).

MRI-to-surgery

The median MRI-to-surgery duration was 200 days during the pre-COVID period, which increased to 225 days in the post-COVID period. The analyses showed a significant difference between the two periods in this regard (P = 0.006) (Figure 3).

## Discussion

The results of the study suggest that delays in MRI could impact the timing of ACL surgery. Specifically, the average and median time from injury to surgery was substantially longer when the time between injury and MRI was longer. In the pre-COVID period, the average time from injury to surgery was 350.8 days and the median time was 287.5 days, with an average of 82 days between injury and MRI. Furthermore, all patients had an MRI scan before an operation, with 35% having the scan before seeing an expert.

The effect of the COVID pandemic on ACL injury prognosis is intricate and multifactorial. Our research demonstrated that the COVID pandemic had a substantial effect on the management of ACL injuries. Although protected knee clinics and surgery were decreased during the pandemic, our research found that the delay from ACL injury to MRI and subsequent surgery was reduced throughout and following the pandemic.

The number of sports injuries has dropped considerably since the pandemic began in March 2020. During COVID-19, the percentage of accidents ascribed to sports decreased, owing primarily to modified or canceled sports seasons [12,13]. In the case of ACL injuries, a Google Trends analysis revealed that from March 2020 to May 2020, the terms ACL reconstruction, ACL reconstruction, and ACL repair were searched less frequently than before the pandemic began, indicating a shift in public interest in this surgery [14].

Aside from its impact on sports accidents, COVID caused many elective operations to be postponed or canceled [15]. This might have caused concerns for patients who had an ACL tear but were unable to receive treatment promptly. Delaying ACL repair further is linked with a greater incidence of meniscal injuries and increased severity of these lesions, whereas operation should be postponed until the effusion subsides and the range of motion of the knee returns [16].

The pandemic has emphasized the importance of managing acute musculoskeletal injuries, including ACL injuries, efficiently and effectively. Our research sheds light on how healthcare systems can adjust to the challenges presented by the epidemic to provide the best possible treatment to patients with ACL injuries. Healthcare workers were able to assess and handle patients with ACL injuries more effectively after implementing telemedicine and remote consultations, decreasing the time from accident to diagnosis and treatment. Our hospital has shown that the time to diagnosis during COVID was two months, while post-COVID was around three months, as compared to 21 months by a study done by Bollen and Scott [6].

**Table 1:** Mean time to reach diagnosis and sample size as per various studies compared to our hospital

No.	Authors	Sample size	Mean time to diagnosis (days)
1	Ball and Haddad, 2010 [17]	100	123
2	Ayre et al., 2017 [3]	120	29
3	Wang et al., 2016 [5]	60	6.35
4	Clifford et al., 2016 [18]	61	115
5	Current study	97	63

Our research had some limitations. First, because our sample size was limited, our results may not be generalizable to other institutions. Second, our research was performed in a unique institution, and the effect of the pandemic on ACL injury management may differ across healthcare systems. Third, we did not look into the influence of the pandemic on patient outcomes; future research should look into whether delays in identification and treatment during the pandemic had any long-term impacts on patient outcomes. This research also lacks a control group, making it impossible to establish whether changes in the management process were caused by the COVID-19 pandemic or if they would have happened anyway over time.

The provision of acute knee clinics and the creation of suitable routes can ensure that patients are evaluated and treated for their injuries as early as possible. The long-term impacts of the COVID-19 pandemic on the treatment of ACL injuries, including patient outcomes and the influence on healthcare systems, should be the focus of future research. Healthcare systems need to keep evolving and innovating to ensure that patients with acute musculoskeletal injuries receive the best possible treatment, even in times of turmoil.

## Conclusions

The COVID-19 pandemic had a significant impact on the management of ACL injuries, with delays in elective knee clinics and surgery potentially leading to delays in the diagnosis and management of ACL injuries. However, our study also showed that the delay from ACL injury to subsequent surgery actually reduced post-pandemic with the provision of acute knee clinics, which is particularly important in the context of associated meniscal injury that can worsen while patients are waiting for surgery. Further studies can be focused on long-term outcomes and concurrent meniscal injuries.
